# Effects of supportive and conflicting interactions with partners and friends on emotions: Do the source and quality of relationships matter?

**DOI:** 10.3389/fpsyg.2022.1020381

**Published:** 2022-12-30

**Authors:** Huiyoung Shin, Sunjeong Gyeong

**Affiliations:** Department of Psychology, Jeonbuk National University, Jeonju, South Korea

**Keywords:** social interactions, relationship source, relationship quality, partners, friends, emotions

## Abstract

This study investigated the independent and interactive effects of supportive and conflicting interactions and overall relationship quality with partners and friends on positive and negative emotions. Data on social interactions and overall relationship quality with partners and friends, and emotions were collected from 717 South Korean adults (*M*_age_ = 47.23; 50.6% male). The results showed that supportive interactions with friends and high relationship quality with partners and friends were associated with enhanced positive emotions, whereas conflicting interactions with partners and friends and low relationship quality with partners were associated with increased negative emotions. In addition, interactive effects of social interactions and overall relationship quality suggested the evidence of reverse buffering. The beneficial effect of friend support on positive emotions was present only when friend conflict was high, and the adverse effect of partner conflict on positive emotions was magnified when individuals perceived high overall relationship quality with their partners.

## Introduction

Close relationships include both social ties that support and social ties that bind and can have both positive and negative impacts on various indicators of emotional well-being (Saphire-Bernstein and Taylor, 2013). Just as individuals can benefit from supportive social interactions by deriving reassurance and comfort from them, such relationships can also be the source of conflict, stress, and strain (Pietromonaco and Rook, 1987). Previous research has emphasized benefits of close and supportive relationships for life satisfaction, happiness, and positive emotions as well as costs of social conflict and tension in loneliness, depression, and negative emotions (Sherman et al., 2011; Chen and Feeley, 2014; Pierce and Quiroz, 2019).

When considering social interactions at a general level, however, it is not possible to ascertain whether the benefits and costs of interactions are because of support and strain occurring within one relationship or across multiple sources of relationships. The ways in which individuals experience support and conflict may depend on the source of relationships (Secor et al., 2017), and the associations between social interactions and emotions could look different when investigated at a general level versus a specific level (Pierce and Quiroz, 2019). Also, the impact of supportive and conflicting interactions on emotions could differ by the overall quality of relationships. The relational context within which supportive or conflicting social interactions take place could be a significant determinant of the effects of support or strain (Cutrona, 1996).

The goal of this study was to examine the independent and interactive effects of supportive and conflicting interactions and overall relationship quality with partners and friends on positive and negative emotions. Considering both positive and negative aspects of social interactions with partners and friends simultaneously would elucidate the relative importance of source-specific support and conflict and potential additive or synergistic influence of social interactions on emotions. Investigating the moderating role of overall relationship quality in the associations of social interactions and emotions could clarify if supportive or conflicting interactions have differential impacts on positive and negative emotions within varying relational context.

### Supportive and conflicting social relationships

Social support serves as a social fund from which individuals can draw emotional, informational, and instrumental assistance (Thoits, 1995). Emotional support involves interaction partners providing caring and understanding. Because emotional support given implies that a person is esteemed and accepted, it has also been referred to as esteem support (Wills, 1985). Information support involves helping individuals understand and cope with stress or challenges, and thus, it has also been referred to as appraisal support or cognitive guidance. Instrumental support refers to providing individuals tangible support, such as needed services or financial aid. Although functions of social support can be distinguished conceptually, they are not usually independent in real life. For instance, interaction partners who demonstrate understanding are also likely to provide information or tangible support (Cohen and Wills, 1985). In this study, we did not discriminate its functions but considered aspects of emotional and informational support.

Social relationships do not always flourish individuals’ emotional well-being. In the process of diverse social interactions, individuals experience social conflict, distress, and strain (Antonucci et al., 2014). Social strain results from different interactions with social partners that cause psychological distress such as resentment and sadness or reservations about the relationships themselves (Rook, 1992). For instance, friends who are too critical or demanding can negatively affect individuals’ emotions, and well-intentioned spouses or partners can be irritating and stress-inducing when their support is either unwanted or represents attempts at control (Antonucci, 1994). Although social strain has received comparatively less attention, accumulative evidence has indicated that conflict and tension have more potent effects on well-being and health than social support (Schuster et al., 1990; Rook, 1997).

Research has suggested that both supportive and conflicting social interactions can occur simultaneously within a certain relationship and exert equivalent effects on positive and negative emotions within their respective domain (the domain-specific effect model; Ingersoll-Dayton et al., 1997). That is, supportive interactions are associated with positive emotions and conflicting interactions are associated with negative emotions. Other research has also demonstrated that there are different types of relationships such as primarily close, primarily problematic, or ambivalent (Fingerman et al., 2004). For example, individuals generally perceived social relationships with spouses and close family members (e.g., parents, children, and siblings) with greater ambivalence than those with friends or acquaintances, whereas individuals classified social relationships with acquaintances as primarily problematic. In general, as individuals feel closer and more intimate toward their relationship partners, they perceive increased ambivalence. Social relationships with long histories are assumed to contribute to mixed sentiments. Social exchanges of affection and shared activities could bring about positive sentiments. Yet, long shared histories, more frequent contact, and increased obligation could also induce stresses, tensions, and irritations (Fingerman and Bermann, 2000; Akiyama et al., 2003).

Empirical evidence underscores that both supportive and conflicting social interactions can be present simultaneously within a specific relationship, but they are independent, representing two distinct domains of social interactions. Additionally, it should be noted that social conflict and tension is qualitatively different from a lack of social support (Okun and Keith, 1998). Social conflict includes negatively charged social interactions, whereas a lack of social support stems from being emotionally disconnected from interaction partners. Therefore, the absence of social support does not equate with the presence of social conflict (Pierce and Quiroz, 2019) because the same interaction partner can provide both support and tension, resulting in ambivalence (Fingerman et al., 2004). Taken together, evidence demonstrates the utility of examining both supportive and conflicting social interactions to elucidate how patterns of the two are differentially associated with individuals’ emotions.

### Independent and interactive effects of support and conflict on emotions

Supportive interactions can have generalized beneficial effects because social networks afford individuals with regular interactions and stability (Thoits, 1983). The main effect model indicates that regular social exchanges with diverse relationship partners provide individuals positive affect, stability and predictability in life, and a recognition of self-worth, all of which can positively contribute to enhanced well-being (Cohen et al., 2000). Considerable evidence supports this main effect model, indicating greater supportive social interactions with relationship partners can lessen loneliness and negative affect (Chen and Feeley, 2014) and foster positive emotions and happiness (Secor et al., 2017). Although the association between supportive interactions and emotional well-being is reciprocal in nature, a growing number of longitudinal studies have demonstrated that supportive interactions predict greater emotional well-being and health (Seeman et al., 1995; Pierce and Quiroz, 2019).

Supportive (and conflicting) interactions can also have interactive effects. The buffering effect model assumes that supportive interactions can mitigate the adverse impact of stress (i.e., conflict and tension) on well-being (Cohen and Wills, 1985). Supportive interactions can assist individuals to work through their emotional reactions to conflict and tension, so that they can be less responsive to social distress (Burleson and Goldsmith, 1996). However, previous research on buffering effects has shown mixed results (see Lincoln, 2000 for a review). Some studies have shown that supportive interactions dampened the distress-producing effects of social conflict (Schuster et al., 1990; Lepore, 1992). Other studies have reported that conflicting interactions reduced the beneficial effects of support on well-being (DeLongis et al., 2004). Evidence for a reverse buffering effect was also reported such that the negative effect of conflicting interactions was magnified as individuals experienced more supportive interactions (Pagel et al., 1987).

Research probing interactive effects has generally considered social interactions within one relationship type (e.g., if supportive interactions with relatives buffered the negative effects of conflict with relatives; Schuster et al., 1990; DeLongis et al., 2004; Holt-Lunstad et al., 2008), with less attention to different relationship sources or cross-relationship buffering effects (e.g., if supportive interactions with friends offset the adverse effects of spousal strain). These limitations could obscure the buffering effects of supportive and conflicting interactions on emotions and might account for the equivocal nature of the findings in the literature. The results for the interactive effects could be different when general level of support is disaggregated and support or strain from different relationship sources are considered simultaneously. Thus, in this study, we consider supportive and conflicting interactions with partners and friends and distinguish between within- and cross-relationship buffering effects to clarify the evidence.

### Moderating effects of overall relationship quality

When individuals experience supportive and conflicting social interactions close together in time, earlier social interactions can provide a proximal relational context for the following interactions. For example, when husbands and wives experienced support and conflict simultaneously, perceived strain interfered with the positive impact of support on emotions by creating shifts in the evaluation of partners’ subsequent actions (e.g., partner’s behavior is interpreted in the context of suspicion; DeLongis et al., 2004). In a similar vein, the overall relationship quality can affect the positive or negative impact of supportive and conflicting interactions on emotions. Perceived overall relationship quality can be considered a more distal relational context for supportive and conflicting social exchanges. Although all close relationships involve some degree of tension and conflict, such strain is usually fleeting and does not taint the intimate relationship on the whole (Fingerman et al., 2004). In this study, we consider such overarching sentiments or perceptions of individuals about their relationships rather than transient emotional reactions.

We anticipate that the perceived relationship quality could play an important role in the associations between supportive and conflicting interactions and positive and negative emotions. In fact, prior research has indicated that spousal support *per se* is not universally beneficial; rather, satisfaction and quality associated with such support is what matters (Holt-Lunstad et al., 2008). Schuster et al. (1990) argued that conflicting interactions between husbands and wives could be perceived as less harmful when such conflicts occur in the relational context of loving and caring relationship. When a husband behaves in an inconsiderate manner, a wife’s interpretation for such unpleasant behavior could be more benign if their relationship is trustworthy and caring overall (Bradbury and Fincham, 1992). Accordingly, the impact of conflict could have less serious impact in the relational context of a generally affectionate relationship.

In contrast, other research has also provided evidence that the detrimental effects of conflict and tension could be particularly salient when their relationships are characterized by intimacy and affection (Rook, 1990). That is, conflicting interactions can have particularly deleterious impact when individuals perceive their relationships as harmonious and trustworthy (Pietromonaco and Rook, 1987) and supportive interactions can be particularly beneficial when it occurs in the context of less supportive relationships (Cutrona, 1996) because such conflict and support could cause strong emotional reactions when they are unexpected. Similar to research considering interactive effects of supportive and conflicting interactions, the literature concerning moderating effects of overall relationship quality in the associations between social interactions and emotions is small and equivocal. Thus, in this study, we investigate the moderating role of overall relationship quality in the associations of supportive and conflicting interactions and positive and negative emotions.

### The present study

The primary aim of this study was to examine the effects of supportive and conflicting social interactions and overall relationship quality with partners and friends on emotions. To elucidate the relative salience of source-specific supportive and conflicting interactions on positive and negative emotions, we considered social support and conflict experienced with partners and friends because prior research has underscored that partners and friends are particularly crucial for individuals’ well-being (Fingerman et al., 2004; Fiori et al., 2006; Birditt and Antonucci, 2007). To enrich understanding of the buffering effects, we investigated within- and cross-relationship buffering effects of source-specific supportive and conflicting interactions on positive and negative emotions. In addition, we examined the moderating roles of overall relationship quality in the associations of social interactions and emotions.

Based on the evidence, we expected that spousal and friend support being associated with enhanced positive emotions, and spousal and friend conflict being associated with heightened negative emotions (Ingersoll-Dayton et al., 1997). In addition, we anticipated both additive and interactive effects of source-specific supportive and conflicting interactions and overall relationship quality in predicting positive and negative emotions. Given that existing research has shown mixed evidence, within- and cross-relationship buffering effects of supportive and conflicting interactions on emotions and the moderating effects of overall relationship quality were largely exploratory in nature, and no specific hypotheses were made.

## Materials and methods

### Participants

We collected data from an online research participant system after receiving approval from the university’s institutional review board in 2021 year. We used stratified probability sampling to obtain a representative sample of different age groups of South Korean adults. Information about the current research was provided prior to starting the survey and informed consent was obtained from all participants. The original sample comprised 1,033 adults representing each decade of adult life span. The current study focused on adults who were married or partnered. This sample consisted of 717 adults (50.6% male, 100% South Korean) aged between 20 and 69 years (*M*_age_ = 47.23, SD = 12.83). About 21% of the participants had less than a high school diploma, the majority (68%) had a college degree, and 11% had a higher than college degree. About one quarter (25%) of the participants reported an annual household income of less than $20,000, and 45% reported an annual household income of more than $40,000. Less than one quarter (19%) of the participants were partnered but not married; the majority (81%) of the participants were married. The majority (74%) of the participants had one or more children. We provided detailed demographic information in Table 1. There were no missing data; all 717 participants responded to all of the items on relationship-specific support and conflict, relationship quality, and positive and negative emotions.

**Table 1:** Demographic information of participants.

	20s	30s	40s	50s	60s	All
						
Sample size (*N*)	93	115	155	180	174	717
Age, mean (SD)	26.08(2.65)	34.81(2.59)	44.12(2.99)	53.18(2.67)	63.34(2.80)	47.23(12.83)
Gender^a^						
Men, *N* (%)	37 (10%)	58 (16%)	82 (23%)	91 (25%)	95 (26%)	363
Women, *N* (%)	56 (16%)	57 (16%)	73 (21%)	89 (25%)	79 (22%)	354
Education^b^						
<High school, *N* (%)	29 (17%)	11 (7%)	23 (16%)	40 (27%)	46 (31%)	148
Some college, *N* (%)	63 (13%)	91 (19%)	118 (24%)	114 (23%)	105 (21%)	491
Graduate school, *N* (%)	1 (1%)	13 (17%)	14 (18%)	26 (34%)	23 (30%)	77
Income^c^						
<$10,000, *N* (%)	14 (10%)	9 (7%)	25 (18%)	51 (37%)	38 (28%)	137
$10,000–$20,000, *N* (%)	6 (13%)	6 (13%)	5 (11%)	10 (22%)	18 (40%)	45
$20,000–$30,000, *N* (%)	25 (26%)	18 (19%)	12 (13%)	18 (19%)	23 (24%)	96
$30,000–$40,000, *N* (%)	21 (18%)	17 (15%)	22 (19%)	19 (17%)	36 (31%)	115
> $40,000, *N* (%)	27 (8%)	65 (20%)	91 (28%)	82 (25%)	59 (18%)	324
Marital status^d^						
Have partner, *N* (%)	84 (61%)	28 (20%)	12 (9%)	11 (8%)	2 (2%)	137
Married, *N* (%)	9 (2%)	87 (15%)	143 (25%)	169 (29%)	172 (30%)	580
Children^e^						
One or more, *N* (%)	5 (1%)	52 (10%)	135 (26%)	165 (31%)	171 (32%)	528
None, *N* (%)	88 (45%)	63 (33%)	20 (11%)	15 (8%)	3 (2%)	189
						

*N* = 717; ^a^Gender is coded 0 = male and 1 = female; ^b^Education is coded 1 = <high school, 2 = some college, 3 = graduate school; ^c^Income is coded 1 = <$10,000, 2 = $10,000–$20,000, 3 = $20,000–$30,000, 4 = $30,000–$40,000, 5 = > $40,000; ^d^Marital status is coded 0 = have partner and 1 = married; ^e^Children is coded 0 = none and 1 = one or more.

### Measures

#### Supportive and conflicting interactions

To assess the extent of supportive and conflicting interactions from partners and friends, we used the positive and negative social support scales (Smith et al., 2013). It consists of 12 items measuring perceived support and conflict for the four relationships, and we used the partner and friend dimension in this study. Smith et al. (2013) reported the Cronbach’s *α*s to be 0.82 and 0.84 for support and 0.79 and 0.81 for conflict for subscales of partners and friends, respectively. Participants responded to each statement using a 5-point scale (1 = *not at all true* and 5 = *very true*). A sample item was, “How much can you rely on them if you have a serious problem?” for support and “How much do they criticize you?” for conflict. The average score was calculated for each subscale, with higher scores indicating greater support and conflict. The scores for Cronbach’s *α*s in this study were 0.86 and 0.82 for support and 0.83 and 0.87 for conflict from partners and friends, respectively.

#### Overall relationship quality

Relationship quality of partners was assessed with the Quality Marriage Index (QMI) developed by Norton (1983) and the adapted version for unmarried couples (Noh et al., 2006). The QMI consists of six items measuring the level of marital satisfaction and has been established to have good internal consistency (*α* = 0.91) and construct validity in prior research (e.g., Maroufizadeh et al., 2019). The adapted version for unmarried couples (e.g., Noh et al., 2006) has also been established to have good internal consistency (*α* = 0.83) and construct validity in prior research. Participants responded to each statement using a 5-point Likert scale (1 = *not at all true*, 5 = *very true*), and sample items included “I really feel like part of a team with my spouse (partner)” and “My relationship with my spouse (partner) is very stable.” The average score was calculated, with higher scores indicating higher levels of relationship satisfaction with one’s partner. Cronbach’s αs of this scale for the current study were 0.94 and 0.93 for married and unmarried couples, respectively.

Relationship quality of friends was assessed with the Friendship Quality Questionnaire developed by Parker and Asher (1993) and revised by Rose (2002). It consists of 12 items measuring the quality of friendships. The scale has been psychometrically strong and has been widely used to evaluate the associations between individuals’ friendships and indicators of emotional and social adjustment (e.g., Rose et al., 2007). Participants responded to each statement using a 5-point Likert scale (1 = *not at all true*, 5 = *very true*). Sample statements included, “We make each other feel important” and “We always tell each other our problems.” The average score was calculated, with higher scores indicates higher levels of friendship satisfaction. Cronbach’s α of this scale was 0.81 in the current study.

#### Positive and negative emotions

Positive and negative emotions were assessed with the Scale of Positive and Negative Experience (SPANE). It is a 12-item measure to assess positive (six items) and negative (six items) feelings (Diener et al., 2010). Because this scale includes general positive and negative emotions, it measures the full range of emotional experiences. Diener et al. (2010) reported Cronbach’s αs of 0.87 and 0.81 for positive and negative emotions, respectively. The construct validity of the scale has been established through the associations with subjective well-being and loneliness. Participants rated how often during the last 4 weeks they had experienced the feelings explained in the statements using a 5-point Likert scale (1 = *very rarely or never*, 3 = *sometimes*, 5 = *very often or always*). Sample statements included “Pleasant” and “Happy” for positive emotions and “Sad” and “Angry” for negative emotions. The sum of scores were calculated, with higher scores indicating higher levels of positive and negative emotions. Scores ranged from 6 to 30 for positive emotions and from 6 to 30 for negative emotions. Cronbach’s αs for this scale in the present study were 0.91 and 0.92 for positive and negative emotions, respectively.

## Results

### Descriptive statistics

Bivariate correlations, means, and standard deviations for source-specific supportive and conflicting interactions, overall relationship quality, and positive and negative emotions are presented in Table 2. Supportive and conflicting interactions with partners and friends were positively associated (*r* = 0.25 and *r* = 0.49 for social support and conflict, respectively). Supportive interactions with partners and friends were positively associated with positive emotions (*r* = 0.31–0.35), and conflicting interactions with partners and friends were positively associated with negative emotions (*r* = 0.33–0.35).

**Table 2:** Bivariate correlations and descriptive statistics for all research variables.

	1	2	3	4	5	6	7	8
1. Partner support	–							
2. Partner conflict	−0.33^**^	–						
3. Friend support	0.25^**^	0.09^*^	–					
4. Friend conflict	0.04	0.49^**^	0.21^**^	–				
5. Partner relationship quality	0.75^**^	−0.53^**^	0.15^**^	−0.12^**^	–			
6. Friendship quality	0.28^**^	−0.08^*^	0.68^**^	−0.03	0.25^**^	–		
7. Positive emotion	0.35^**^	−0.08^*^	0.31^**^	0.07^*^	0.41^**^	0.33^**^	–	
8. Negative emotion	−0.18^**^	0.35^**^	0.01	0.33^**^	−0.33^**^	−0.12^**^	−0.51^**^	–
Range	1–5	1–5	1–5	1–5	1–5	1.33–4.83	6–30	6–30
*M*	3.59	2.31	3.11	1.80	3.71	3.29	20.40	14.49
SD	0.92	0.94	0.86	0.87	0.95	0.58	5.21	5.57

*N* = 717; ^*^p < 0.05, ^**^p < 0.01.

### Independent effects of source-specific support and conflict on emotions

**Figure 1:**
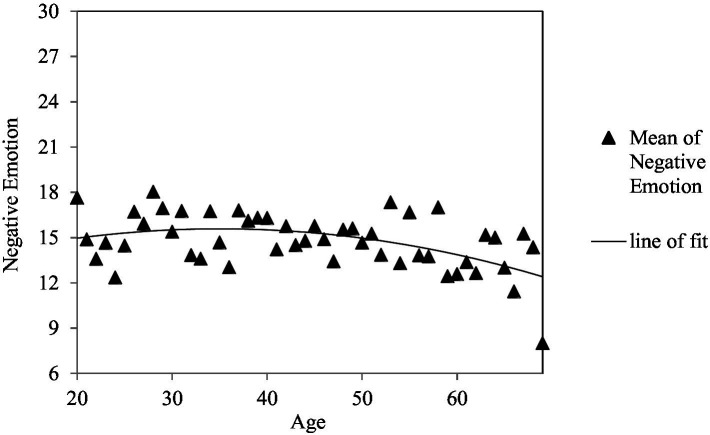
Age^2^ distribution of negative emotion.

First, we examined the relative contributions of supportive and conflicting interactions with partners and friends in predicting positive and negative emotions, controlling for participants’ age, age^2^, gender, education, and income by running a series of hierarchical multiple regressions^1^. Age^2^ was considered in the model to capture the curvilinear pattern of positive and negative emotions across the adult lifespan (Baird et al., 2010). As shown in Table 3, the final model accounted for 29% and 23% of the variance in positive and negative emotions, respectively. Of several demographic variables, age, age^2^, and income were significant predictors. Older adults reported lower levels of negative emotions (see Figure 1) and those who had higher income reported higher levels of positive emotions and lower levels of negative emotions. Taking into account such effects, supportive interactions with friends and high relationship quality with partners and friends were associated with enhanced positive emotions, whereas conflicting interactions with partners and friends and low relationship quality with partners were associated with increased negative emotions.

**Table 3:** Associations of social interactions, overall relationship quality, and emotions.

	Positive emotion		Negative emotion	
	B	SE	B	SE
***Covariates***				
Age	0.02	0.01	−0.09^***^	0.02
Age^2^	0.01	0.01	0.01^***^	0.01
^a^ Gender	0.41	0.36	−0.18	0.40
^b^ Education	0.45	0.32	0.02	0.35
^c^ Income	0.31^**^	0.11	−0.24^*^	0.12
***Social interaction main effects***				
Partner support	−0.36	0.30	0.57	0.33
Partner conflict	0.31	0.27	0.80^**^	0.29
Friend support	1.10^***^	0.29	−0.27	0.32
Friend conflict	0.24	0.25	1.38^***^	0.28
Partner relationship quality	2.48^***^	0.32	−1.81^***^	0.35
Friendship quality	1.06^*^	0.42	−0.59	0.46
***Within-relationship buffering effects***				
Partner support × Partner conflict	0.25	0.30	−0.03	0.34
Friend support × Friend conflict	1.03^**^	0.35	0.05	0.39
***Cross-relationship buffering effects***				
Partner support × Friend conflict	0.22	0.26	0.10	0.29
Friend support × Partner conflict	−0.17	0.22	0.24	0.24
***Relationship quality moderation***				
Partner support × Partner relationship quality	−0.10	0.20	−0.02	0.22
Partner conflict × Partner relationship quality	−0.70^*^	0.29	0.21	0.32
Friend support × Friendship quality	0.13	0.28	0.17	0.31
Friend conflict × Friendship quality	−0.56	0.52	−0.72	0.57
*R* ^2^	0.29		0.23	
*F*	15.26^***^		14.22^***^	

When the interaction terms were added to the models, they explained 2.2% additional variance (*p* < 0.01) for positive emotion; B = unstandardized regression coefficient; ^a^Gender is coded 0 = male and 1 = female; ^b^Education is coded 1 = < high school, 2 = some college, 3 = graduate school; ^c^Income is coded 1 = <$10,000, 2 = $10,000–$20,000, 3 = $20,000–$30,000, 4 = $30,000–$40,000, 5 = > $40,000. ^*^p < 0.05, ^**^p < 0.01, ^***^p < 0.001.

### Interactive effects of source-specific support and conflict, and relationship quality

**Figure 2:**
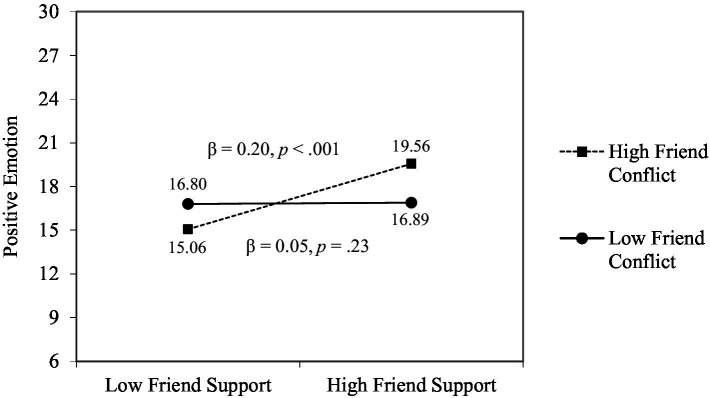
Significant interactions of friend support and friend conflict.

**Figure 3:**
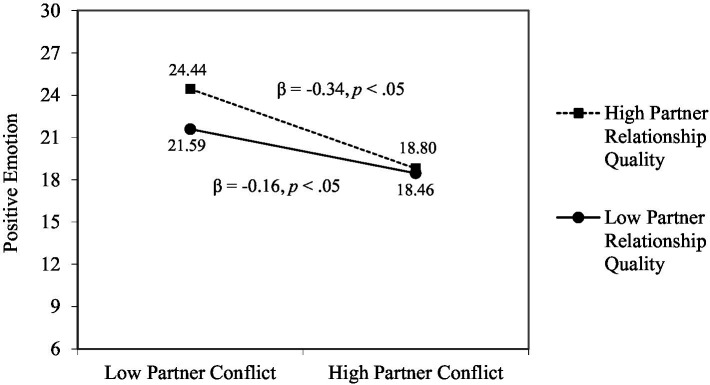
Significant interactions of partner conflict and partner relationship quality.

To clarify within- and cross-relationship buffering effects, we included two-way interaction terms related to supportive and conflicting interactions within each relationship (i.e., partner support × partner conflict, friend support × friend conflict) and across relationship domain (i.e., partner support × friend conflict, friend support × partner conflict). Also, to investigate the moderating role of overall relationship quality in the associations of supportive and conflicting interactions and positive and negative emotions, we included two-way interaction terms related to source-specific supportive and conflicting interactions and overall relationship quality for each relationship (e.g., partner support × partner relationship quality). To reduce problems of multicollinearity, all research variables were centered by subtracting their means (Frazier et al., 2004).^2^ As shown in Table 3, we found a significant within-relationship buffering effect and moderating effect of overall relationship quality. The beneficial effect of friend support on positive emotion was present only when friend conflict was high (see Figure 2), and the adverse effect of partner conflict on positive emotions was magnified when individuals perceived high overall relationship quality with their partners (see Figure 3).

## Discussion

The main purpose of this study was to investigate how supportive and conflicting social interactions with partners and friends are related to individuals’ emotions. Our focus was on the independent and interactive effects of source-specific supportive and conflicting interactions and overall relationship quality on positive and negative emotions. Our results lend support to our approach of considering both positive and negative aspects of social interactions with partners and friends and underscore that investigating both source and quality of relationships are important in elucidating different implications of social interactions on emotions.

### Independent effects of support and conflict, and relationship quality on emotions

As anticipated, supportive and conflicting social interactions and overall relationship quality with partners and friends had significant independent effects on positive and negative emotions. We found that friend support and high relationship quality with partners and friends were more strongly associated with enhanced positive emotions than reduced negative emotions, and conflicts with partners and friends were robustly associated with heightened negative emotions. Overall, evidence substantiated the domain-specific effect model (Ingersoll-Dayton et al., 1997) regarding associations between social interactions and emotions, which posited that positive and negative social interactions exert comparable effects but within their respective domains. That is, supportive interactions (e.g., reassurance, confiding, and respect) have stronger effects on positive emotions (e.g., feeling excited and proud), whereas conflicting interactions produce more intense negative emotions (e.g., feeling nervous and irritable).

It was interesting to find that only friend support was associated with increased positive emotions, whereas the associations between overall relationship quality and positive emotions were significant for both partners and friends. Findings indicated that overall relationship quality was more important predictor of positive emotions than fleeting supportive and conflicting social interactions. The partner relationship is generally characterized as an intimate, close, long-lasting, and singularly important relationship. Most people usually turn to their partners to share positive as well as negative life events. Thus, overarching sentiments about partnership (e.g., trustworthy and reliable) and overall quality associated with support is more consequential than brief emotional experiences (Holt-Lunstad et al., 2008). Yet, long shared histories, frequent contact, and increased obligation and expectation could still induce stresses and irritations (Akiyama et al., 2003; Fingerman et al., 2004). In contrast, friends provide support to each other when faced with life stressors or when a partner is not available. As peers of the same historical cohort with accumulated shared experiences, friends can be a critical and reliable source of support (Antonucci et al., 2010). Whereas support provided by one’s partner could be considered as obligatory and a partner relationship is generally difficult to sever, friends generally choose to provide support and maintain relationships. Such achieved nature of friendships could play an important role in individuals’ emotional well-being.

Our findings showed that supportive and conflicting interactions with partners and friends, and their overall relationship quality had additive influence on positive and negative emotions. Conflicting interactions with partners and friends were additionally associated with negative emotions, and relationship quality with partners and friends additively contributed to positive emotions. Also, the fact that overall quality of friendship had beneficial effects on positive emotions over and above those of friend support points to the utility and value of considering both source and quality of relationships. Taken together, our findings suggest that the positive or detrimental effects conferred by different relationships could be additive and highlight that both supportive and conflicting interactions can have critical implications for emotions.

### Within- and cross-relationship buffering effects of support and conflict on emotions

Results showed that friend support interacted with friend conflict such that the beneficial effect of friend support on positive emotions was present only when individuals experienced high friend conflict. This result is consistent with a reverse buffering effect hypothesis, which has been proposed and tested by Okun and Keith (1998), suggesting that negative social interactions could be more impactful when interpreted against the backdrop of positive social interactions. That is, when individuals often experience supportive or conflicting interactions with certain relationship partners, unusual conflict or support could have a particularly strong impact because of its rarity (Rook, 1990). The fact that friend support had a significant effect on positive emotions only when individuals experienced high friend conflict suggests that positive social interactions can have more potent effects when they occur less frequently because they can have more salience. In this study, we considered such buffering effects related to supportive and conflicting interactions both within a relationship and across relationship domains, but we only found a significant within-relationship buffering effect. Given that research that examines both within- and cross-relationship buffering effects of support and conflict is limited, future studies should use a different population to investigate this issue and replicate our results.

### Moderating effects of overall relationship quality

The perceived relationship quality can be conceptualized as a more distal and relatively stable relational context for supportive and conflicting social interactions. Consistent with prior evidence (Gallo et al., 2003; Holt-Lunstad et al., 2008), we found that overall relationship quality with partners and friends was a significant predictor of positive and negative emotions while controlling for supportive and conflicting interactions. In addition to the independent effects, overall relationship quality with partners moderated the association between partner conflict and positive emotions such that the adverse effect of partner conflict on positive emotions was magnified when individuals perceived high relationship quality with their partners. This counter-intuitive finding suggests the complicated nature of partner relationships. Similar to our finding on the within-relationship buffering effect, conflicting interactions with partners could have particularly deleterious effects when the overall partner relationship is characterized as harmonious, because such conflict and tension can invoke strong emotional reactions when they are unexpected and have rarity (Pietromonaco and Rook, 1987). This feature of partner relationship does not preclude other beneficial effects from being present though. Overall relationship with partners could affect the success or failure of the adaptive resolution processes after conflicting interactions. Those who display a positive sentiment toward their partners would be more successful in relationship repair processes after conflict (Carrère and Gottman, 1999; Sangalang and Gee, 2012). Future studies should continue to examine how day-to-day supportive and conflicting interactions and overall sentiments or judgments of their partners are related to one another and affect individuals’ emotional well-being.

### Limitations and future directions

Apropos of these findings, interesting research to investigate in the future is the effect of personality in the relational contexts within which supportive and conflicting interactions take place. Research has shown that nature and characteristics of social relationships reflect individuals’ personalities (Sarason et al., 1990; Asendorpf and Wilpers, 1998). Given personality is a relatively stable trait characteristic, it is likely that the same type of individuals who have supportive interactions with friends are also more likely to cultivate healthy marriages. Furthermore, given that individuals experience both supportive and conflicting interactions simultaneously (Ingersoll-Dayton et al., 1997), they could seek positive interactions in marital relationships to offset negative impacts of family relationships in which they may be involved.

Our research mostly focused on the source and quality of relationships in examining the effects of supportive and conflicting interactions on emotions. However, the relative significance of different relationships could change over time due to several age-related life events. As individuals progress through old age, support from direct family members could become more crucial and affect their emotions (Carstensen, 1995). Also, cultural norms could play a role in the social dynamics and interactions. For instance, social interactions between parents and partners and their joint effects on emotions could differ between Western and non-Western populations (Ryan et al., 2014). In many Asian countries, filial piety is considered as one of the central social norms and the parent–child relationship continues to be interdependent over the life course (Epstein et al., 2005). Asian adults’ obligation to respect and care for their parents could affect the nature of the partner relationship. We are not aware of any studies that considered filial piety as a potential predictor of social interaction and emotions. Such studies could clarify the complex pathways by which partner relationships influence individuals’ emotions across cultures.

It should be noted that this study was cross-sectional in research design. Thus, the directionality of effects should be interpreted with caution. Further, our measure of supportive and conflicting interactions had substantial overlap with the measure of overall relationship quality. Future research should use other well-validated measures that could better capture the immediate and proximal social interactions. Using other methodological approaches, such as interviews or structured diaries could provide a different perspective. Survey data may not be equipped to assess day-to-day social interactions that could be driving the results found between individuals. Qualitative assessments may help to specify these mechanisms. Lastly, effect sizes of our results across the board were rather small. There are clearly other factors that contribute to individuals’ emotions beyond social interactions and overall relationship quality. However, it has long been argued that effects of social interactions are small because they are ongoing and happening on a daily basis so dramatic effects are not likely to be detected in short-term studies. Nonetheless, small effects are meaningful because they are cumulative and over time contribute to developmental processes. Thus, an examination of social interaction processes contributing to modest effects in individuals’ positive and negative emotions may still be important.

## Conclusion

Despite these limitations, the current research contributes unique information to the field in that it elucidates what specific aspects of social relationships are critically related to emotions. Supportive interactions with friends had a robust effect on positive emotions, whereas conflicting interactions with both partners and friends had stronger effects on negative emotions than on positive emotions. Given this, it can be assumed that positive day-to-day social interactions are critical for individuals’ emotional well-being. In addition, overall relationship quality was more important predictor of positive emotions than fleeting supportive and conflicting interactions. Although the adverse effect of conflict on emotions could be magnified when individuals experience satisfying relationships, overarching sentiments and overall quality associated with support could be more consequential than fluctuating and transient emotional experiences. Collectively, the current results underscore that specifying social interactions with different relationship sources and considering relationship quality are important to clarify their significant roles as predictors and moderators in the link between social interactions and emotions.

## Data availability statement

The datasets used in this study are not readily available because all data are treated with confidentiality. Descriptive information can be provided upon request.

## Ethics statement

The studies involving human participants were reviewed and approved by Jeonbuk National University’s Institutional Review Board. The patients/participants provided their written informed consent to participate in this study.

## Author contributions

HS conceived of the study, helped analyses and interpretation of the data, and drafted the manuscript. SG did analyses and interpreted the data. All authors contributed to the article and approved the submitted version.

## Funding

The research received funding from the Brain Korea 21 Fourth Project of the Korea Research Foundation (Jeonbuk National University, Psychology Department no. 4199990714213).

## Conflict of interest

The authors declare that the research was conducted in the absence of any commercial or financial relationships that could be construed as a potential conflict of interest.

## Publisher’s note

All claims expressed in this article are solely those of the authors and do not necessarily represent those of their affiliated organizations, or those of the publisher, the editors and the reviewers. Any product that may be evaluated in this article, or claim that may be made by its manufacturer, is not guaranteed or endorsed by the publisher.

## Footnotes

^1^We used both the entire sample (i.e., married and partnered) and subsample (i.e., married) to run a series of hierarchical multiple regressions, and compared the differences in pattern of results. Pattern of results remained the same. Thus, we reported results based on the entire sample.

^2^We checked the Variance Inflation Factors (VIF) and tolerance values for each independent variable to make sure that there were not multicollinearity issues in our regression models. It is expected that VIF values smaller than 10 and tolerance values greater than 0.01 indicates the absence of a multicollinearity problem (Kline, 2005). In this study, VIF values of our research variables were 1.02–3.73, which were much below the acceptable threshold. The tolerance values of our research variables were 0.27–0.98, which were above the acceptable threshold. Thus, it was confirmed that there was not a multiple dependency problem among independent variables in the model.
